# The trend in cesarean myomectomies and the risk of obstetrical complications in Korea

**DOI:** 10.1186/s12884-022-04674-3

**Published:** 2022-05-03

**Authors:** Min Jeong Kim, Kyungeun Lee, Jae Young Park, Ji Hye Jo, In Yang Park

**Affiliations:** 1 Department of Obstetrics and Gynecology, College of Medicine, Bucheon St. Mary’s Hospital, The Catholic University of Korea, Seoul, Republic of Korea; 2 Department of Obstetrics and Gynecology, College of Medicine, Seoul St. Mary’s Hospital, The Catholic University of Korea, Seoul, Republic of Korea

**Keywords:** Leiomyoma(s), Cesarean myomectomy, Cesarean section, Pregnancy outcome, Risk factors

## Abstract

**Background:**

To evaluate pregnancy outcomes and the risk of adverse obstetrical outcomes of cesarean myomectomy (CM) compared with cesarean section (CS) only, and to investigate the trend of surgeons in choosing CM.

**Methods:**

A retrospective cohort study was performed on all patients who underwent CS complicated by leiomyoma at two university hospitals between January 2010 and May 2020. All patients were categorized into the CM (341 women) or CS-only (438 women) group. We analyzed the demographic factors, obstetric factors, surgical outcomes, and possible risk factors for adverse outcomes between the two groups.

**Results:**

Women who underwent CS only were significantly more likely to have a previous myomectomy and multiple leiomyoma history than women who underwent CM. The gestational age at delivery and pregnancy complications were significantly higher in the CS-only group. The mean size of the leiomyomas was larger in the CM group than in the CS-only group (5.8 ± 3.2 cm vs. 5.2 ± 3.1 cm, *P* = 0.005). The operation time and history of previous CS and preterm labor were higher in the CM group. The leiomyoma types differed between the two groups. The subserosal type was the most common in the CM group (48.7%), and the intramural type was the most common in the CS-only group. Patients in the CM group had fewer than three leiomyomas than those in the CS-only group. Preterm labor and abnormal presentation were relatively higher in the CM group than in the CS-only group, concerning leiomyoma presence. There were no significant differences in the preoperative and postoperative hemoglobin levels. The size of the leiomyoma (odds ratio [OR] = 1.162; 95% confidence interval [CI]: 1.07–1.25; *P* < 0.001) and operation time > 60 min (OR = 2.461; 95% CI: 1.45–4.15) were significant independent predictors of adverse outcomes after CM.

**Conclusions:**

CM should be considered a reliable and safe approach to prevent the need for another surgery for remnant leiomyoma. Herein, surgeons performed CM when uterine leiomyomas were large, of the subserosal type, or few. Standardized treatment guidelines for myomectomy during CSs in pregnant women with uterine fibroids should be established.

## Background

Uterine leiomyomas are the most common benign tumors of female reproductive organs, with a prevalence of 20–25% [1]. In South Korea, the number of women who visited medical clinics for uterine leiomyomas increased from 340,191 in 2016 to 514,260 in 2020 [2]. According to several studies, the prevalence of leiomyoma during pregnancy has been reported to range from 2 to 5% [3–5]. Most leiomyomas are asymptomatic during pregnancy. Abdominal pain, pelvic pressure, and vaginal bleeding may occur in symptomatic pregnant women. Various obstetric complications such as preterm delivery, miscarriage, fetal growth retardation (FGR), and failure of vaginal delivery may also occur in pregnant women with leiomyomas [3].

The use of robot-assisted or laparoscopic myomectomy for uterine leiomyomas has also increased. In particular, when uterine leiomyomas are present in young women of childbearing age or when myomectomy has been performed, pregnancy-related problems and various complications may occur. Preterm labor, placental problems, and uterine rupture are particularly serious obstetric problems; therefore, research on pregnancy, uterine leiomyoma, and myomectomy is very important.

Although uterine rupture related to myomectomy is extremely rare, the relationship between myomectomy during pregnancy and outcomes is very important in the management of pregnant women by obstetricians. In addition, the effect of myomectomy on subsequent pregnancies is not fully known. The most alarming complication is uterine rupture. There are insufficient data on uterine rupture during pregnancy after myomectomy in South Korea. In one retrospective study conducted at a single medical center, uterine rupture occurred in 3 (0.6%) out of 523 pregnant women who underwent prior myomectomy [6]. Although uterine rupture during pregnancy is rare, it can be life-threatening for the mother and the fetus. The American College of Obstetricians and Gynecologists recommends that women who have had a prior myomectomy that breached the endometrial cavity undergo cesarean delivery to avoid the possible complication of uterine rupture [7].

Generally, cesarean myomectomy (CM) is discouraged because of the risk of postpartum complications such as hemorrhage, fever, and ileus [8]. However, when myomectomy is performed concurrently with cesarean section (CS), the burden of an additional surgery under general anesthesia is reduced. In recent decades, an increasing number of studies have demonstrated the safety and feasibility of CM [9, 10].

Our study aimed to evaluate the outcomes of CM vs. CS alone in women with uterine leiomyomas, evaluate the risk factors for adverse outcomes in women undergoing CM, and analyze why surgeons choose CM.

## Methods

**Fig. 1:**
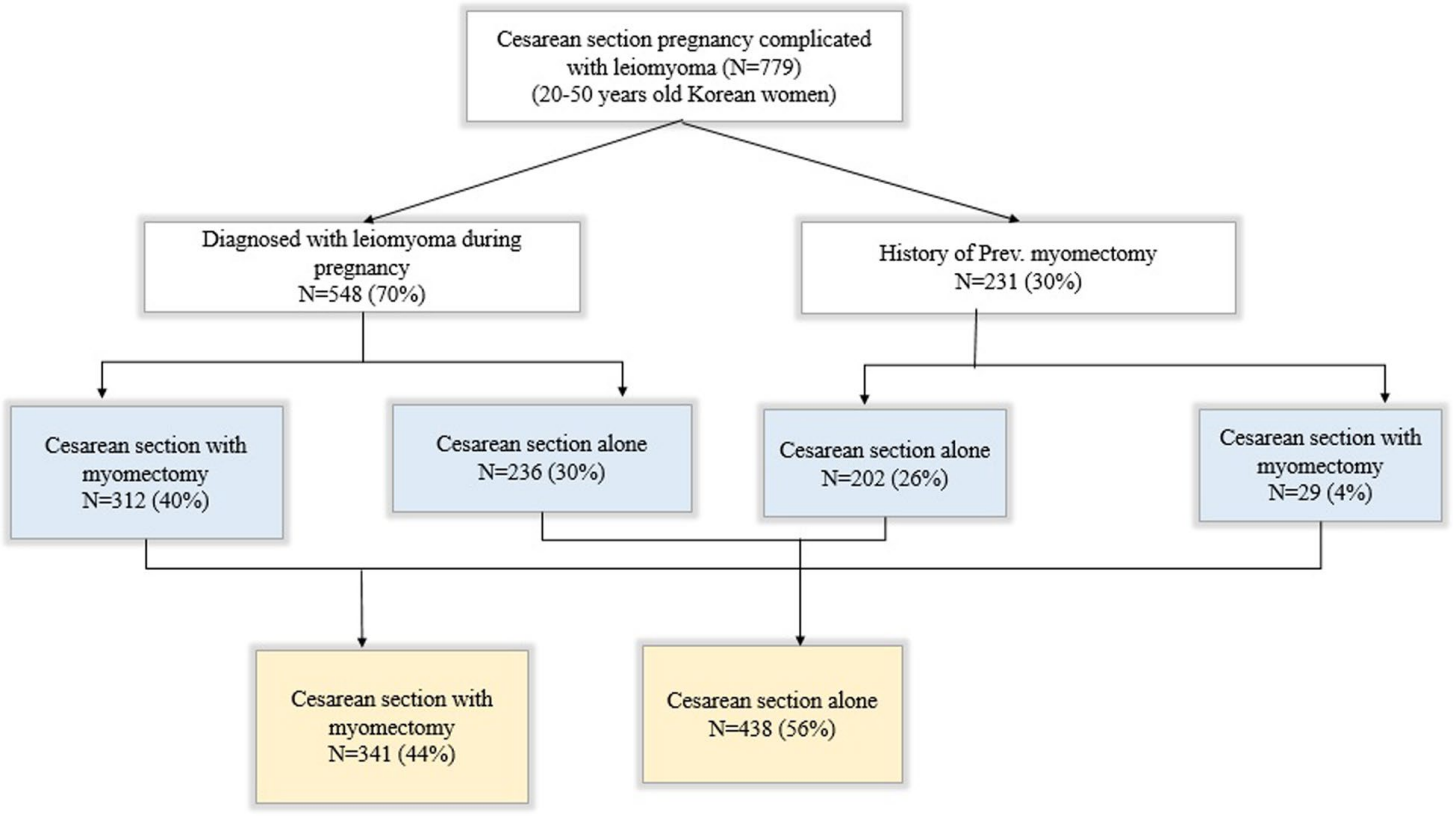
Flow chart for the selection of the studies

This retrospective cohort study was conducted on cesarean deliveries complicated by leiomyomas at two university hospitals between January 2010 and May 2020. This study was approved by the Institutional Review Board of the Catholic University of Korea for the use of anonymized patient data for medical research (XC20WIDI0093). Data were collected from our electronic health record database, and administrative permission was obtained for the data used in our research. The study was conducted in accordance with the guidelines of the Declaration of Helsinki, and the rights of all the participants were protected. The study included women diagnosed with leiomyoma detected by antenatal ultrasound or with a history of myomectomy (Fig. 1).

Maternal demographic characteristics included maternal age, body mass index (BMI), parity, gestational age at delivery, multiple pregnancies, fetal presentation, neonatal birth weight, Apgar score of the neonates, fetal presentation, FGR, and assisted reproduction techniques. The leiomyoma characteristics were evaluated, and history of the previous uterine myomectomy and preterm labor were included. Leiomyomas were categorized according to the size, type, and number. The size of the leiomyoma was determined from pathology reports. If there were no pathology reports, information on the size or type of the leiomyoma was obtained from the operative notes or antenatal ultrasound.

We compared the outcomes of women who underwent CM with those who underwent CS only without myomectomy. The measured outcome parameters included operation time, emergency operation, hemoglobin (Hgb) changes between preoperative and postoperative values, transfusion after surgery, and intraoperative and postoperative complications, such as postpartum hemorrhage, intrauterine balloon tamponade insertion, and hysterectomy.

The possible risk factors for adverse outcomes in women who underwent CM were analyzed using multivariate regression analysis. The evaluated factors were related to the location of the leiomyoma, the number of leiomyomas, the size of the leiomyoma, neonatal birth weight, placental problems, preeclampsia, and operation time > 60 min.

### Statistical analysis

All statistical analyses were performed using SAS version 9.4 (SAS Institute, Cary, NC, USA). Continuous data are presented as mean ± standard deviation and categorical data as numbers and percentages. P-values were calculated using the chi-square test or Fisher's exact test for categorical variables and the t-test or Wilcoxon rank-sum test for continuous variables. Statistical significance was set at *P* < 0.05. We calculated odds ratios (ORs) and 95% confidence intervals (CIs) using logistic regression models to assess the independent predictors of adverse outcomes of CM.

## Results

From January 2010 to May 2020, 779 patients who underwent CS and were either diagnosed with leiomyoma (*n* = 548) or had a history of myomectomy (*n* = 231) were included. There were 438 women in the CS-only group and 341 women in the CM group (Table 1). The average age of the patients was 34.8 ± 3.6 years and 34.8 ± 3.7 years in the CS-only and CM groups, respectively.

**Table 1:** Epidemiological characteristics between the CS-only and CM groups

	CS-only group (*n* = 438)	CM group (*n* = 341)	*P*-value
Maternal age (years)	34.8 ± 3.6	34.8 ± 3.7	0.918^*^
Height (cm)	161.1 ± 5.2	161.6 ± 5.2	0.224^*^
Weight (kg)	68.9 ± 10	69.4 ± 11.1	0.570^*^
BMI (kg/m^2^)	26.3 ± 3.4	26.4 ± 3.8	0.758^*^
Nulliparous (%)	385 (87.9)	302 (88.6)	0.776
Abortion, mean ± SD	0.4 ± 0.8	0.4 ± 0.8	0.901
Gestational days at delivery (day)	261.5 ± 20	259.2 ± 21.7	0.021
Singlet/twin			0.142
Single (%)	420 (95.9)	319 (93.6)	
Twin (%)	18 (4.1)	22 (6.5)	
Birth weight of baby (g)	2891.7 ± 685.8	2818.4 ± 734.4	0.101
Apgar score at 5 min < 7 (%)	82 (18.7)	81 (23.8)	0.087
Presentation of the fetus			0.056
Cephalic (%)	374 (85.8)	281 (82.4)	
Abnormal presentation (%)	62(14.25)	60 (17.6%)	
FGR of the baby (%)	38 (8.7)	36 (10.6)	0.374
Operation time (min)	44.5 ± 18.2	52.5 ± 23.8	< 0.001
Emergency operation (%)	133 (30.4)	110 (32.3)	0.572

Values are numbers (percentages) or means (standard deviations) for categorical variables

*P-values* are calculated using the chi-square test or Fisher's exact test for categorical variables and t-test^*^ or Wilcoxon rank sum test for continuous variables

*CS* Cesarean section, *CM* Cesarean myomectomy, *BMI* Body mass index, *FGR* Fetal growth retardation

The indications for CS included previous myomectomy (*n* = 209, 26.8%), fetopelvic disproportion (*n* = 189, 24.3%), fetal distress (*n* = 77, 9.9%), abnormal fetal presentation (breech, transverse position; *n* = 77, 9.9%), pathological placental presentation (placenta previa, low-lying placenta, or placental abruption; *n* = 58, 7.4%), known leiomyoma (n = 63, 8.1%), history of a previous CS (*n* = 38, 4.9%), FGR, twins, preeclampsia, and preterm labor.

Table 1 shows the demographic characteristics of the study population. There were differences in the gestational age at delivery (261.5 ± 20 days [CS-only group] vs. 259.2 ± 21.7 days [CM group]; *P* = 0.021) and the operation time (44.5 ± 18.2 min [CS-only group] vs. 52.5 ± 23.8 min [CM group]; *P* < 0.001) between the two groups. Although there were no statistically significant differences between the groups in terms of fetal presentation, the rate of abnormal presentations was slightly higher in the CM group (17.6% vs. 14.25%; *P* = 0.056). There were no significant differences in maternal age, maternal BMI, parity, number of fetuses, or emergency operations. In addition, no significant differences were detected in the Apgar scores of the neonates.

The characteristics related to uterine leiomyoma and operative outcomes are shown in Table 2. A lower percentage of patients in the CM group underwent a previous myomectomy (8.5% vs. 46.1%; *P* < 0.001), whereas there was a higher percentage of patients with previous CS or preterm labor in the CM group (7.3% vs. 3.4%; *P* = 0.014, 32.3% vs. 22.25%; *P* = 0.002). The mean leiomyoma size in the CM group was 5.8 ± 3.2 cm, which was significantly larger than that of the CS-only group (*P* = 0.005). In addition, the types of leiomyomas were different. Intramural leiomyoma was most common in the CS-only group (59.5%), whereas subserosal leiomyoma was most common in the CM group (48.7%). More patients in the CM group (90.9%) had fewer than three leiomyomas than those in the CS-only group (72.4%), which had a higher percentage of patients with multiple leiomyomas. Complications related to pregnancy, especially placental problems, preeclampsia, and oligohydramnios, occurred significantly more often in the CS-only group (24.4%) than in the CM group (10.6%). Pathological examination revealed degeneration in 54.6% of leiomyomas after CM. One patient was later diagnosed with smooth uterine muscle of uncertain malignant potential, and total hysterectomy was performed. There were no statistically significant differences in terms of postoperative complications such as postpartum hemorrhage, total hysterectomy, or insertion of intrauterine balloon tamponade.

**Table 2:** Characteristics related to uterine leiomyoma and obstetrical factors between the CS-only and CM groups

	CS-only group (*n* = 438)	CM group (*n* = 341)	*P*-value
History of previous CS (%)	15 (3.4)	25 (7.3)	0.014
History of previous myomectomy (%)	202 (46.1)	29 (8.5)	< 0.001
History of preterm labor (%)	97 (22.2)	110 (32.3)	0.002
Size of the leiomyoma (cm), mean ± SD	5.2 ± 3.1	5.8 ± 3.2	0.005
Type of the leiomyoma (%)			< 0.001^†^
Intramural leiomyoma	122 (59.5)	139 (41.5)	
Subserosal leiomyoma	70 (34.2)	163 (48.7)	
Submucosal leiomyoma	4 (2)	5 (1.5)	
Mix type leiomyoma	7 (3.4)	28 (8.4)	
Cervical leiomyoma	2 (1)	-	
Number of leiomyomas (%)			< 0.001
Number < 3	165 (72.4)	301 (90.9)	
Number ≥ 3	63 (27.6)	30 (9.1)	
IVF (%)	391 (89.3)	294 (86.2)	0.195
Transfusion during CS (%)	22 (5)	16 (4.7)	0.832
Complication of CS (PPH + Backri, adhesion, inverted C/sec, T/H) n (%)	4 (3.2)	7 (2.1)	0.328
Complication of pregnancy (placental problem [low lying, pl.previa, pl.abruptio, pl.accreta], preeclampsia, oligohydramnios) (%)	107 (24.4)	36 (10.6)	< 0.001

Values are numbers (percentages) and means (standard deviations) for categorical variables

*P*-values are calculated using the chi-square test or Fisher's exact test† for categorical variables

*CS* Cesarean section, *CM* Cesarean myomectomy, *IVF* In vivo fertilization, *PPH*, Postpartum hemorrhage, *C/sec* Cesarean section, *T/H* Total hysterectomy, *pl.previa* Placenta previa, *pl.abruptio* Placental abruption, *pl.accreta* Placenta accreta

No significant difference was observed between the preoperative and postoperative Hgb levels between the groups (Table 3). The initial preoperative Hgb was 12.1 ± 1.3 mg/dL in the CS-only group and 12 ± 1.3 mg/dL in the CM group. The postoperative day 1 Hgb was 11 ± 1.4 mg/dL in the CS-only group and 10.9 ± 1.5 mg/dL in the CM group.

**Table 3:** Hematologic changes between the CS-only and CM groups

	CS-only group (*n* = 438)	CM group (*n* = 341)	*P*-value
Initial preop Hgb (mg/dL)	12.1 ± 1.3	12 ± 1.3	0.313^*^
Pod #1 Hgb (mg/dL)	11 ± 1.4	10.9 ± 1.5	0.761^*^
Pod #3 Hgb (mg/dL)	10 ± 1.3	9.9 ± 1.4	0.311^*^

Values are means (standard deviations) for categorical variables

*P*-values are calculated using the chi-square test, t-test^*^, or Wilcoxon rank sum test for continuous variables

*CS* Cesarean section, *CM* Cesarean myomectomy, *preop* Preoperative, *Hgb* Hemoglobin, *Pod #1* Postoperative day 1, *Pod #3* Postoperative day 3

Multiple logistic regression analysis was performed to identify the risk factors for adverse outcomes after CM, such as blood transfusion, decrease in Hgb levels, or other postoperative complications. The leiomyoma size (OR = 1.162; 95% CI: 1.07–1.25; *P* < 0.001) and operation time > 60 min (OR = 2.461; 95% CI: 1.45–4.15; *P* = 0.001) were significant independent predictors of adverse outcomes after CM (Table 4).

**Table 4:** Independent risk factors of adverse outcomes of CM

	OR	95% CI	*P*-value
Birth weight of the baby	1.000	1.000–1.000	0.432
Size of the leiomyoma	1.162	1.077–1.253	< 0.001
Number of leiomyomas > 3	2.078	0.950–4.548	0.067
Location of the leiomyoma (anterior)	1.228	0.745–2.023	0.42
Location of the leiomyoma (posterior)	0.666	0.314–1.414	0.29
Placental problem (low lying, pl.previa, pl.abruptio, pl.accreta)	0.241	0.030–1.929	0.18
Preeclampsia	0.891	0.273–2.910	0.848
Operation time > 60 min	2.461	1.458–4.154	0.001

Outcome: Transfusion or complication of CS or change in the hematological result (a decrease of > 3 mg/dL in the hemoglobin level)

ORs are calculated using logistic regression

*CM* Cesarean myomectomy, *OR* Odds ratio, *CI* Confidence interval, *pl.previa* Placenta previa, *pl.abruptio* Placental abruption, *pl.accreta* Placenta accrete

## Discussion

This study aimed to evaluate the factors that determine complications during pregnancy associated with uterine leiomyoma and provided important information for managing women’s health. Herein, the surgeons performed CM when uterine leiomyomas were large, of the subserosal type, or few. There were no significant differences in the postoperative Hgb levels and postoperative complications, such as the incidence of postpartum hemorrhage, postoperative insertion of intrauterine balloon tamponade, or hysterectomy. Based on these results, CM appears to be safe.

The mean gestational age at delivery was significantly lower in the CM group, which might have been due to the increased contractility of the myometrium with the presence and mass effect of the leiomyoma [11]. Similarly, the incidence of preterm labor increased significantly in the CM group in our study. In addition, although not statistically significant, there was a higher rate of abnormal fetal presentation in the CM group, suggesting that the presence of leiomyomas affected fetal malpresentation [12, 13].

Our data suggest a significant difference (8 min) in the duration of surgery between the groups. In a previous report, the operation time was 4.94 min longer in the CM group than in the CS-only group, which was not a significant difference [14]. In another study, the operation time in the CM group was 15 min longer than that in the CS-only group, which was a significant result [11].

The number of patients with a history of myomectomy was significantly higher in the CS-only group than in the CM group. This may be because a history of myomectomy itself is an indication for CS. This may have been because patients who underwent a previous myomectomy were likely to have fewer or no leiomyomas, so only CS was performed. The number of patients with a history of CS was significantly higher in the CM group. The probable reason for this was to prevent additional surgery for remnant leiomyomas in the future.

Generally, CM is controversial and not routinely recommended. This is because CM tends to increase the rate of intraoperative or postoperative hemorrhage and, in the worst case, leads to hysterectomy and pelvic adhesions due to bleeding. A meta-analysis was conducted in 2017, examining 19 studies and comparing 2,301 patients who underwent CM with those who underwent CS only [15]. This study reported that the group that underwent CM had a greater decrease in Hgb levels and needed more blood transfusions (mean difference in Hgb 0.25 mg/dL, 95% CI: 0.06–0.45; risk of transfusion OR: 1.41, 95% CI: 0.96–2.07). In contrast, in a retrospective cohort study conducted in 2019, there were no differences in terms of the average decrease in Hgb levels or blood transfusion rates between patients who underwent CM and those who underwent CS only [16]. Similarly, in our study, we did not find any significant differences between the groups in terms of a decrease in the Hgb levels or blood transfusion rates. In addition, postoperative complications, such as the incidence of postpartum hemorrhage, postoperative insertion of intrauterine balloon tamponade, or hysterectomy, also showed no significant differences. Therefore, CM is a reliable and safe approach and seems to prevent future operations.

Many factors should be carefully considered before performing CM, such as the patient’s condition, location of the leiomyoma, emergency status of the surgery, and the surgeon’s skill. In particular, the surgeon’s skill and preference for CM are likely to play an important role because CM can be associated with operative complications.

Pregnancy-related uterine rupture after myomectomy is a dangerous complication in both the mother and the fetus. Gambacorti et al. reported that labor after myomectomy was associated with a 0.47% risk of uterine rupture [17]. According to a study by Koo et al., uterine rupture during pregnancy occurred in only three (0.6%) of 523 patients who underwent laparoscopic myomectomy [6]. This study concluded that laparoscopic myomectomy is a safe surgical option for women who desire future pregnancy. However, studies on the correlation between CM and uterine rupture are limited. Additional research is required for a better understanding of this relationship.

Standardized indications for CM have not been defined yet. As derived from our results, the characteristics of leiomyomas in the CM group were mostly the subserosal type, singular or fewer than three in number, or large in size. This seems to be a result of surgeons considering the risk benefits of CM to avoid risks during surgery. According to a study by Zhao et al. [16], the presence of a leiomyoma > 5 cm and birth weight > 4,000 g were important risk factors for postpartum hemorrhage ≥ 1,000 mL in pregnant women with leiomyomas during CS, whereas the location and type of leiomyoma had little effect. Kwon et al. reported that a large size (> 8 cm) and lower segmental position of the leiomyoma were significant risk factors for intraoperative hemorrhage during CM [18].

Our study has some limitations. First, in pregnant women with leiomyoma, some underwent CM and others underwent CS alone. CM is not routinely performed in South Korea. Rather, obstetricians tend to be reluctant to perform CM for the above-mentioned reasons. Most patients in the CM group included in our study also had an unplanned CM. In most cases, CM was performed when it was unavoidable due to accessibility or when the location and size of the leiomyoma were such that they were easy to remove. This may have produced biased results. This study was initiated based on the idea that standard treatments and indications for CM are needed as the number of cases increases. Therefore, it is necessary to establish the indications for CM through a well-designed prospective study. Second, our study included only short-term outcomes. Since our study includes relatively recent data, the amount of data and the study period are insufficient to observe long-term outcomes. Identifying long-term outcomes would strengthen the evidence for the safety and usefulness of CM.

## Conclusions

CM should be considered as a safe approach for pregnant women with leiomyomas. In the present study, the independent risk factors affecting adverse outcomes after CM were the size of the leiomyoma and the duration of surgery. The larger the leiomyoma and the longer the surgery, the higher the risk of adverse outcomes. Therefore, a prospective study on the effectiveness of CM is needed. Thus, objective data on the surgeon’s skill and safety of CM should be accumulated. Therefore, a new clinical guideline for CM should be developed in future studies.

## Data Availability

All authors had full access to the data and materials. The data are available from the authors upon reasonable request.

## Abbreviations

BMI: Body mass index
CI: Confidence interval
CM: Cesarean myomectomy
CS: Cesarean section
Hgb: Hemoglobin
FGR: Fetal growth retardation
OR: Odds ratio
